# Design of Pt-Sn-Zn Nanomaterials for Successful Methanol Electrooxidation Reaction

**DOI:** 10.3390/ma16134617

**Published:** 2023-06-27

**Authors:** Dragana Milošević, Sanja Stevanović, Dušan Tripković, Ivana Vukašinović, Vesna Maksimović, Vladan Ćosović, Nebojša D. Nikolić

**Affiliations:** 1 Department of Ecology and TechnoEconomics, Institute of Chemistry, Technology and Metallurgy, University of Belgrade, Njegoševa 12, 11000 Belgrade, Serbia; dragana.milosevic@ihtm.bg.ac.rs; 2 Department of Electrochemistry, Institute of Chemistry, Technology and Metallurgy, University of Belgrade, Njegoševa 12, 11000 Belgrade, Serbia; dusan@ihtm.bg.ac.rs; 3 Department of Mathematics and Physics, Faculty of Agriculture, University of Belgrade, Nemanjina 6, 11080 Belgrade, Serbia; ivanavu@agrif.bg.ac.rs; 4 Vinča Institute of Nuclear Science—National Institute of the Republic of Serbia, University of Belgrade, 11000 Belgrade, Serbia; 5 Department for Materials and Metallurgy, Institute of Chemistry, Technology and Metallurgy, University of Belgrade, Njegoševa 12, 11000 Belgrade, Serbia; vlada@tmf.bg.ac.rs

**Keywords:** platinum-based catalysts, nanoparticles, microwave polyol synthesis, methanol electrooxidation

## Abstract

This work highlights the potential for the synthesis of new PtSnZn catalysts with enhanced efficiency and durability for methanol oxidation reaction (MOR) in low-temperature fuel cells. In this research, PtZn and PtSnZn nanoparticles deposited on high surface area Vulcan XC-72R Carbon support were created by a microwave-assisted polyol method. The electrochemical performances of synthesized catalysts were analyzed by cyclic voltammetry and by the electrooxidation of adsorbed CO and the chronoamperometric method. The physicochemical properties of obtained catalysts were characterized by transmission electron microscopy (TEM), thermogravimetric (TGA) analysis, energy dispersive spectroscopy (EDS) and by X-ray diffraction (XRD). The obtained findings showed the successful synthesis of platinum-based catalysts. It was established that PtSnZn/C and PtZn/C catalysts have high electrocatalytic performance in methanol oxidation reactions. Catalysts stability tests were obtained by chronoamperometry. Stability tests also confirmed decreased poisoning and indicated improved stability and better tolerance to CO-like intermediate species. According to activity and stability measurements, the PtSnZn/C catalyst possesses the best electrochemical properties for the methanol oxidation reaction. The observed great electrocatalytic activity in the methanol oxidation reaction of synthesized catalysts can be attributed to the beneficial effects of microwave synthesis and the well-balanced addition of alloying metals in PtSnZn/C catalysts.

## 1. Introduction

Polymer electrolyte membrane fuel cells (PEMFCs) use the chemical energy of hydrogen or small organic molecules such as methanol, ethanol or formic acid to cleanly and efficiently produce electricity [1,2,3,4,5]. PEMFCs are exceptional due to the range of potential applications; they can supply electricity for applications across many sectors, including transportation, and for large systems such as residential and industrial buildings and can also act as small power devices in laptop computers and mobile phones. Fuel cells have many benefits in comparison to conventional technologies, which are used combustion energy since they provide higher efficiencies than combustion engines and produce electrical energy from the fuel with an efficiency of 60%. In comparison to combustion engines, fuel cells do not release air pollutants that create smog. However, cost, efficiency and lifetime are still the most challenging issues in the industry of fuel cells [5].

Platinum is the most expensive component of PEMFCs, so there is imperative to develop catalysts with increased catalyst activity and, at the same time, reduce the content of expensive platinum through alloying with less expensive metals such as Sn, Mo, Zn, Fe, Co, Au, Bi, Ni, etc. [6,7,8,9,10,11,12,13,14,15,16].

To improve catalysts’ efficiency and durability, the focus of this research will be on innovative synthesis strategies of PtZn and PtSnZn catalysts with enhanced efficiency and durability for the methanol oxidation reaction. The PtSn catalyst is one of the most efficient binary platinum catalysts for the methanol electrooxidation reaction. Also, Sn is a widely available and inexpensive metal, and its promoting effect is expressed through a bi-functional and electronic effect. The bifunctional effect represents the facilitated oxidation of highly adsorbed species that blocks platinum surfaces (produced during the methanol oxidation reaction) in the presence of another element and consequently leads to the inactivation of the Pt catalyst as well as the reduction of activity for MOR. Namely, the added Sn leads to the formation of oxygen species that are necessary for the oxidation of highly adsorbed carbonaceous intermediates at much lower potentials compared to platinum. According to many researchers, the inclusion of Sn activates water at a significantly lower potential than the presence of Pt [17] and forms hydroxyl-adsorbed species (OH_ads_) on the catalyst surface. The produced OH_ads_ species promote the elimination of CO_ads_, which is an unwanted product of the methanol electrooxidation reaction since CO is strongly adsorbed on active platinum sites. In the electronic effect, the effect of the added element is manifested through its influence on the electronic structure of platinum. As a result of the electron exchange that occurs by alloying the added element with platinum, platinum’s affinity for CO adsorption decreases. In the case of added Sn in platinum catalysts, the electronic properties of Pt are altered, resulting in a reduction in the adsorption capacity of strongly adsorbed molecules like CO [18,19,20]. Zn is also an interesting alloying metal due to its electronic structure. Namely, Zn has an empty 4(s,p) orbital and a fully filled 3d orbital, which gives it the ability to act as an electron donor or acceptor, depending on the other metal. In the case of alloying with Pt, Zn induces electron relocalization within Pt from its 5d to 6(s,p) orbitals, which reduces the energy of the Pt 5d band in the PtZn alloy [21,22,23]. As a consequence, the adsorption bond strength of CO is weakened. The density functional theory investigations (DFT) show that the presence of Zn atoms on the platinum surface can intensify the adsorption of CH_2_OH* intermediated species. As a consequence, the reaction-free energy of the rate-determining step of the methanol oxidation reaction is reduced, and hence the electrocatalytic activity of the PtZn catalyst is improved. Also, there are reports from DFT calculations that predict that MOR on PtZn-i catalysts prefers to follow a “non-CO” reaction pathway (* + CH_3_OH → CH_2_OH* → CH_2_O* → CH_2_O* + OH* → H_2_COOH* → HCOOH* → HCOO* → * + CO_2_) [24,25].

Based on all of these considerations, the combining of Pt-Sn-Zn nanoparticles synthesized by the microwave-assisted polyol method may lead to an even better methanol oxidation reaction in comparison to PtSn and PtZn nanocatalysts. In this work, carbon-supported nanoparticles, PtZn and PtSnZn catalysts, synthesized by a microwave-assisted polyol method, were tested for methanol electrooxidation reaction (MOR). To the best of our knowledge, this reaction by PtSnZ/C catalyst prepared by a microwave irradiation method has not been examined so far.

## 2. Materials and Methods

### 2.1. Preparation of Catalysts

Stable PtZn and PtSnZn nanoparticles were synthetized by the microwave-assisted polyol method. To obtain the PtZn/C catalyst, 0.5 mL of 0.05 M H_2_PtCl_6_ solution was mixed with 0.5 mL 0.05 M ZnSO_4_ solution and 25 mL of ethylene glycol in a 100 mL Erlenmeyer flask, while the same amount of platinum and ethylene glycol were mixed with 0.25 mL 0.05 M ZnSO_4_ and 0.25 mL 0.05 M SnCl_2_ solutions to obtain the PtSnZn/C catalyst. In order to adjust pH~12, 0.8 M NaOH was added dropwise. The prepared solutions were mixed for 30 min under magnetic stirring and then placed in the microwave oven. The reduction reaction was carried out by microwave irradiation at 700 W for 90 s. After microwave heating, the colloidal solutions were mixed with 20 mL of Vulcan XC-72R carbon water suspension and 150 mL 2 M H_2_SO_4_ for 3 h, prompting the homogeneous deposition of the colloids on the carbon support. The final suspension was filtered by a vacuum pump, and the solid residue was rinsed with high-purity water (Millipore, 18 MΩ cm, Darmstadt, Germany). The solid products were dried for 3 h in a N_2_ atmosphere at 160 °C. Metallic loading for both catalysts was adapted to 20 mass %.

### 2.2. Characterization of the Catalyst

All catalysts were examined by the thermogravimetric (TGA) and differential thermal (DTA) analyses performed in the range of 30–1000 °C range on an SDT Q600 TGA/DSC instrument (TA Instruments, New Castle, DE, USA). The heating rates were 20 °C min^−1^, and the sample masses of the analyzed samples were 7.1520 mg for the PtZn/C catalyst and 9.9570 mg for the PtSnZn/C catalyst. The furnace atmosphere was composed of air with a flow rate of 100 cm^3^ min^−1^.

The chemical composition of the samples was obtained by a scanning electron microscope (SEM) Tescan VEGA TS 5130 MM supplied with energy-dispersive X-ray spectroscopy (EDS) detector INCAPentaFET-x3, Oxford Instruments, High Wycombe, United Kingdom). The samples were acquired using 20 kV acceleration voltages from at least five different regions for each catalyst sample with the aim of obtaining the average values.

For detailed characterization of the morphology of obtained catalysts, transmission electron microscope (TEM) JEM-1400 with an accelerating voltage of 120 kV was used. The samples were made by ultrasonically dispersed PtZn/C and PtSnZn/C catalysts in water. A drop of the suspensions was deposited onto the carbon-coated copper grid. The average size of obtained catalyst nanoparticles and their size distributions were calculated using ImageJ software. More than 100 separated particles were counted for that purpose.

Phase analysis of the PtZn/C and PtSnZn/C catalysts was performed using an X-ray diffractometer (XRD) Rigaku Ultima IV, Japan (Tokyo, Japan), with CuKα1 radiation (CuKα = 0.154178 nm). A single crystal silicon plate was used as a sample holder. The scanning rate was 2°/min, and the 2θ range was from 10° to 90° with a step of 0.02°. The PDXL2 2.0.3.0 software, with reference to the diffraction patterns available in the International Center for Diffraction Data (ICDD), was used for phase identification and data analysis. The structural parameters were obtained from the crystal structure database (ICSD) (for Pt ICSD 03-065-2868).

### 2.3. Electrochemical Measurements

All of the electrochemical measurements were carried out at room temperature. The electrochemical cell was made of three-electrode-compartment: Pt wire was used as the counter electrode, and a bridged saturated calomel electrode (SCE) as reference one. The electrocatalyst ink for the working electrode was composed of 2 mg of the synthetized catalyst in a suspension of 1 mL water and 50 µL of 5% aqueous Nafion solution. The obtained solution was prepared in an ultrasonic bath. The solution was ultrasonically stirred for 1 h, and after that, 10 μL of the solution (catalyst loading of 20 g/cm^2^) was placed onto the top of the glassy carbon substrate (working electrode) and dried ~2 h at room temperature.

The electrocatalytic activity of synthetized catalysts was examined in 0.5 M H_2_SO_4_ + 0.5 M CH_3_OH solution. Methanol was added to the basic electrolyte solution while maintaining the electrode potential at −0.2 V. The methanol electrooxidation reaction was investigated in as-prepared catalysts whose surfaces were without any previous treatment in the supporting electrolyte (0.5 M H_2_SO_4_).

Catalyst stability was examined by chronoamperometry and potential long-term cycling. The chronoamperometric curves (current-time curves) were recorded after immersion of the freshly prepared electrodes in the solution at −0.2 V for 2 s. After that, the potential was raised to 0.2 V. The electrodes were kept at that potential for 30 min.

The electrochemical surface area (ECSA) of catalysts was calculated from CO_ads_ stripping voltammetry. CO gas was introduced in the electrochemical cell with supporting electrolyte for few minutes in order to obtain saturated 0.5 M H_2_SO_4_ solution. After that, CO was adsorbed at the electrode surface while keeping the electrode potential at −0.2 V vs. SCE for 15 min. After adsorption, the electrodes were moved into the cell with 0.5 M H_2_SO_4_, and the CO_ads_ was oxidized in an anodic scan (sweep rate 50 mV/s). The first three voltammograms were recorded in order to confirm the completeness of the CO oxidation. The ECSA was calculated from CO stripping considering that the theoretical charge for removal of CO monolayer is 420 μC cm^−2^. The specific catalyst activities were normalized in relation to the values found for the real surface area and platinum mass loading. Methanol and CO_ads_ oxidation were investigated on separate catalyst surfaces with comparable basic voltammograms.

All solutions were made using Merck p.a. reagents and high-purity water. Before each experiment, the electrolytes were purged with nitrogen. AUTOLAB potentiostat/galvanostat PGStat 128 N (ECO Chemie, Utrecht, The Netherlands) was employed for electrochemical measurements.

## 3. Results and Discussion

### 3.1. Characterization of PtZn/C and PtSnZn/C Catalysts

Recognized as catalysts with great potential for methanol electrochemical oxidation, PtZn/C and PtSnZn/C catalysts have been successfully synthesized by the microwave synthesis process. All catalysts were analyzed by the TGA/DTA analysis, XRD and EDX examination. The results for TGA and EDX are presented in Table 1.

TGA analysis was used to determine the amount of metals in the catalyst powder. TGA curves for PtZn and PtSnZn catalysts are given in Supplementary Materials (Figures S1 and S2). The derivative thermogravimetric curves, which expressed mass variation as a function of temperature, reveal 27.68 wt.% and 22.87 wt.% for PtZn/C and for PtSnZn/C catalysts. Slightly higher values of the residual mass compared to the nominal value can be attributed to the presence of ZnO in the catalyst, which can increase the reaction extent during the heating procedure [26]. EDX analysis revealed that the obtained ratio of metals (expressed in mass percentages) in the synthesized catalysts deviates slightly from the initial calculations, i.e., the desired values. The Pt:Zn ratio for the PtZn/C catalyst is 87 wt.%:13 wt.% while Pt:Sn:Zn ratio is 70 wt.%:21 wt.%:9 wt.% for PtSnZn/C catalyst (Supplementary Materials Figures S3 and S4).

**Figure 1:**
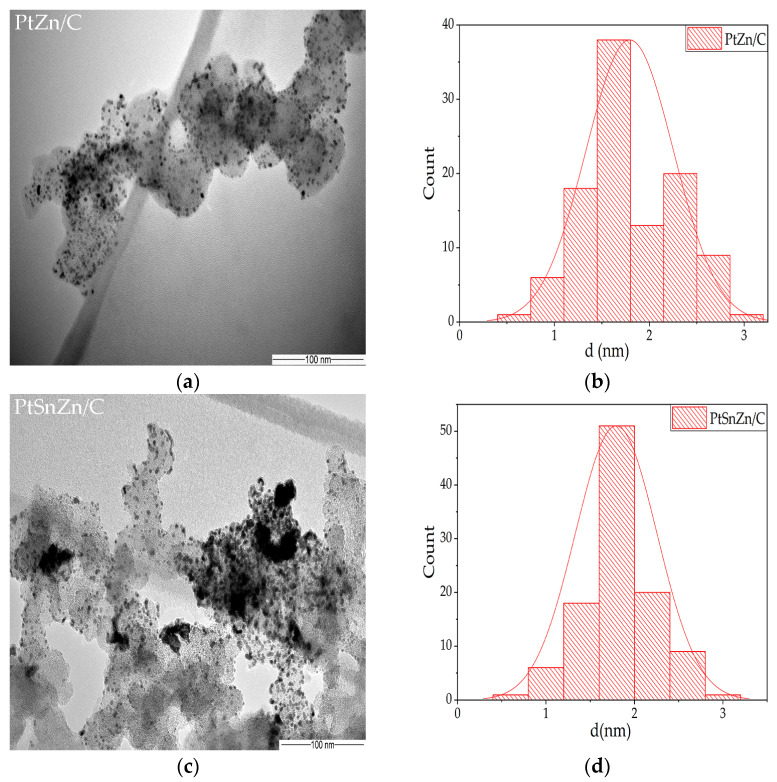
TEM images of PtZn/C (**a**) and PtSnZn/C catalysts (**c**). Particle size distribution of PtZn/C (**b**) and PtSnZn/C catalysts (**d**).

In order to determine the size of the particles in the synthesized catalysts, a TEM analysis was performed. All catalysts’ particle sizes were fairly similar, according to the TEM examination. Figure 1 displays the typical images and related histograms of particle size distribution. According to statistical analysis, the observed catalyst particles have diameters of 1.63 ± 0.3 for PtZn/C and 1.8 ± 0.4 for PtSnZn/C catalyst (particle size distribution histogram shown in Figure 1b,d).

**Figure 2:**
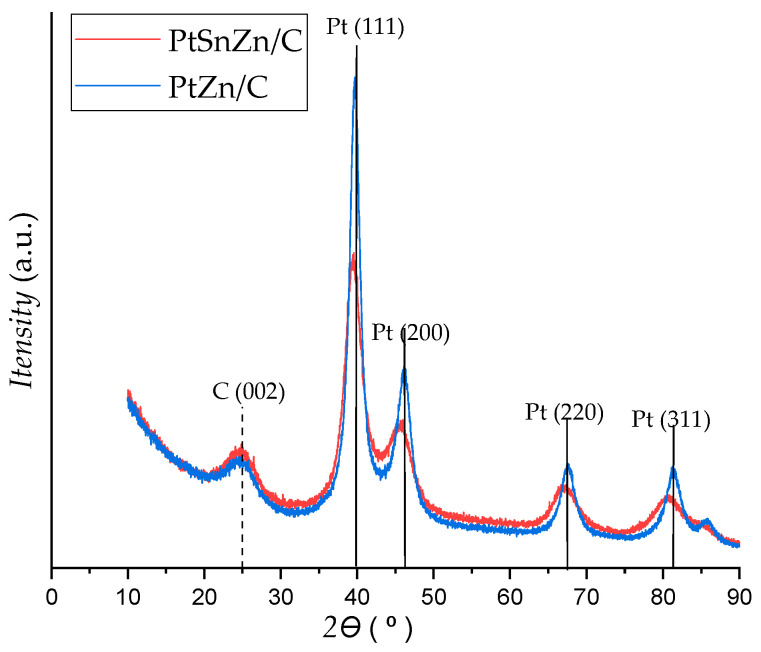
XRD patterns of PtZn/C and PtSnZn/C catalysts (corresponding planes for fcc Pt crystal structure are presented with black lines).

XRD analysis of PtZn/C and PtSnZn/C catalysts is presented in Figure 2. From the analysis of the XRD spectra, the main characteristic peaks of the face-centered cubic (fcc) Pt crystalline structure (JCPDS 04-0802) for both catalysts can be clearly seen. From the XRD diagram, it can also be observed that there are slight shifts in the diffraction peaks for PtSnZn/C and PtZn/C catalysts in comparison to the characteristic diffraction peaks of platinum. These are the peaks: (111) at 40°, (200) at about 46°, (220) at about 67.5° and (311) at 81.5°, and they are marked by a black vertical line in the XRD diagram. The diffraction peak at 25° originates from the substrate, i.e., the hexagonal structure of Vulkan XC–72 R, and it is indicated by a black dashed line on the XRD diagram.

Slight shifts of the characteristic peaks towards smaller 2θ values indicate the incorporation of a larger tin atom into the crystal lattice of platinum. Due to the slightly larger atomic radius of tin (0.14 nm) than the atomic radius of platinum (0.139 nm), there is a change in the crystal structure of the platinum lattice, i.e., its expansion. No peak for pure zinc or zinc oxide can be observed on the XRD diagram for the PtZn/C catalyst. However, the presence of Zn in the synthesized catalyst cannot be excluded, considering that Zn can be present in an amorphous form. This is supported by the fact that TGA analysis did not show metal loss during synthesis, while EDS analysis showed the presence of Zn in the synthesized catalyst (Table 1).

### 3.2. Electrochemical Performance of the Catalysts

**Figure 3:**
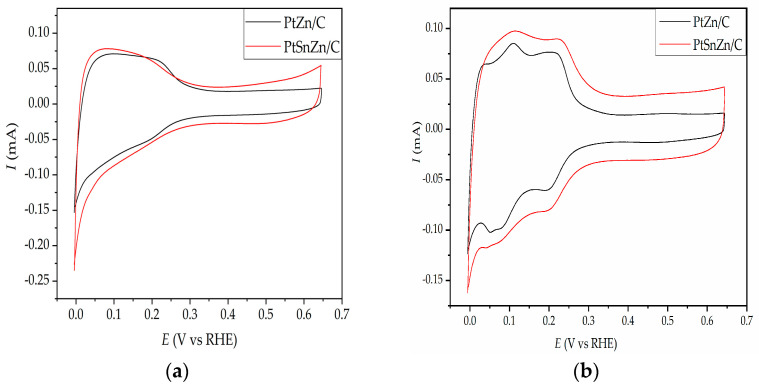
Cyclic voltammograms of (**a**) 1st cycle and (**b**) 30th cycle of PtZn/C (black line) and PtSnZn/C catalysts (red line) in 0.5 M H_2_SO_4_, v = 50 mV/s.

The cyclic voltammetry experiments were used to determine the electrochemical behavior of PtZn/C and PtSnZn/C catalysts. Cyclic voltammetry experiments are done on untreated, as-prepared catalysts since Sn and Zn dissolve at potentials over 0.4 V versus SCE, while at those potentials, their oxide is stable [23,27]. The basic voltammograms of all catalysts are illustrated in Figure 3.

The voltammograms of both catalysts are in agreement with voltammograms that have been reported in the literature for platinum catalysts synthesized in a similar procedure [28,29]. In the potential region from −0.25 V to 0.1 V, which represents a hydrogen adsorption/desorption region, the typical peaks related to (110) and (100) locations of polycrystalline Pt particles [30,31] are not distinguishable. This is a result of the adsorption of various carbon and oxygen species on the catalyst surfaces during drying in the air atmosphere of the catalyst on the glassy carbon electrode and is also a result of displaying the first cyclic voltammogram of PtZn/C and PtSnZn/C catalysts. After the 30th cycle, both catalysts show a well-developed hydrogen adsorption/desorption region (Figure 3b).

### 3.3. Oxidation of Adsorbed CO

As an extremely structurally sensitive reaction, oxidation of CO_ads_ was carried out not only to calculate catalysts’ electrochemically active surface area but also with the purpose of establishing structure-activity relationships for resistance to CO tolerances of the examined catalysts surfaces. The interpretation of the oxidation of CO on the platinum catalyst through the Langmuir-Hishelvod mechanism, which takes place through several steps, has been established. In the first step, oxygen species are formed on the electrode surface, and this can be represented by the following equation [32]:

(1) $$H_{2}O\;⇆\;{\mathrm{OH}}_{\mathrm{ads}}\;+\;H^{+}\;+\;e^{-}$$

The next step represents a chemical reaction between the adsorbed species [33]:

(2) $${\mathrm{CO}}_{\mathrm{ads}}\;+\;{\mathrm{OH}}_{\mathrm{ads}}\;\to \;{\mathrm{COOH}}_{\mathrm{ads}}$$

In the final step, there is a charge transfer [30]:

(3) $${\mathrm{COOH}}_{\mathrm{ads}}\;\to \;{\mathrm{CO}}_{2}\;+\;H^{+}\;+\;e^{-}\;$$

**Figure 4:**
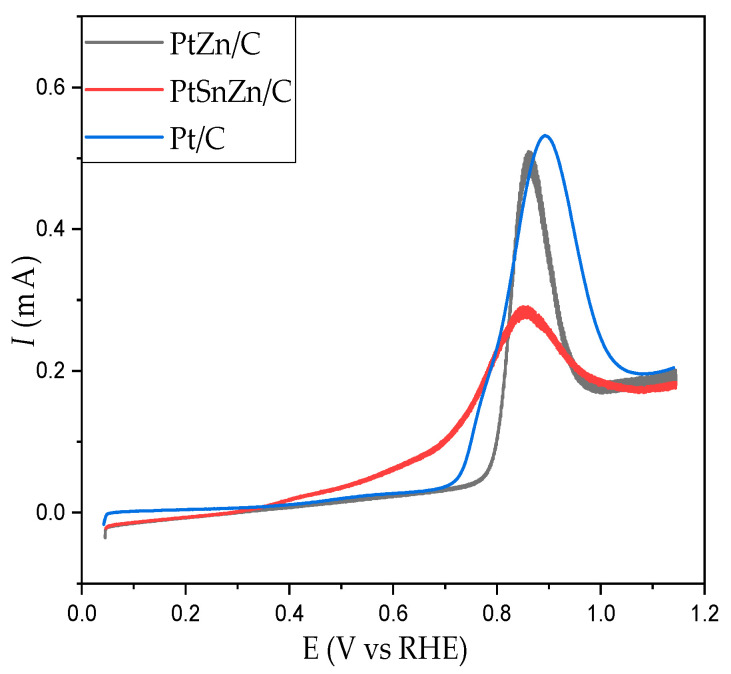
Oxidation voltammograms (stripping voltammograms) of adsorbed CO for as-prepared PtZn/C (black line), PtSnZn/C (red line) and Pt/C (blue line) catalysts in 0.5 M H_2_SO_4_, v = 50 mV/s.

As can be seen from Figure 4, the oxidation of CO_ads_ for Pt/C catalysts is represented by a sharp peak at 0.89 V vs. RHE. Although pure Sn and Zn do not react with CO, the rate of CO oxidation on the Pt catalyst is enhanced by alloying Pt with these metals [6,20,34]. Stripping curves for CO_ads_ from PtZn/C and PtSnZn/C catalysts surfaces are also presented in Figure 4. Oxidation of adsorbed CO proceeds through a sharp peak at 0.86 V vs. RHE for the PtZn/C catalysts, while for the PtSnZn/C catalyst, CO oxidizes through a broad potential area with the same maximum potential (0.86 V). Examining the oxidation of adsorbed CO on the PtSnZn/C catalyst (Figure 4), we can notice that the onset of the reaction starts by ~0.4 V earlier compared to the other two catalysts. The presence of tin contributes to the increase of activity at low potentials in the case of PtSnZn/C catalysts, which is in accordance with the literature data [35,36,37]. Namely, due to the significant difference in electronegativity between platinum and tin, there is a transfer of electrons from tin to platinum, which induces an increase in the electron density around the Pt sites [38]. As a result, the adsorption of CO and OH on the surface of the catalyst decreases. Since CO does not bind to tin, while OH prefers to be adsorbed on tin, the beginning of oxidation moves to negative potential values, that is, to values at which OH is adsorbed on tin [38]. This increase in the oxidation rate in bimetallic PtSn catalysts is explained in the literature by the bifunctional and electronic effect of tin.

Determination of the real surface area is very important in order to normalize the activities of different electrocatalysts to the same number of reactive surface sites. The total charge transfer was determined by integrating the stripping voltammograms in the potential region, and the calculated electrochemically active surface areas (ECSAs) are summarized in Table 2. The ECSA was calculated assuming that the theoretical charge for the removal of the CO monolayer is 420 µC/cm^2^. This assumption comes from many literary works, among which is an overview paper [39].

### 3.4. Methanol Oxidation

The activity of the as-prepared PtZn/C and PtSnZn/C catalysts for methanol oxidation was obtained from potentiodynamic measurements (Figure 4a,b, respectively).

MOR’s fundamental mechanisms principles tell that the ideal reaction includes the transfer of six electrons and the direct oxidation of the methanol molecule to CO_2_. The overall methanol oxidation reaction is as follows [41]:

CH_3_OH + H_2_O → CO_2_ + 6H^+^ + 6e^−^ (4)

Nevertheless, MORs includes more steps, such as methanol adsorption and dehydrogenation, and, at the same time, the accumulations of carbon-containing intermediates, the first being the generation of carbon monoxide (CO_ads_). In the actual process, the intermediate product CO_ads_ is always present; it covers the catalysts’ surface leading to the blocking of active sites and reducing the effectiveness of the catalyst. Oxidation of CO_ads_ begins when oxygen-containing species (OH_ads_) are sufficiently generated by the interaction of water with platinum to finally form CO_2_ in a Langmuir–Hinshelwood reaction mechanism (Equation (5)) [42]:

CO_ads_ + OH_ads_ → CO_2_ + H^+^ + e^−^. (5)

**Figure 5:**
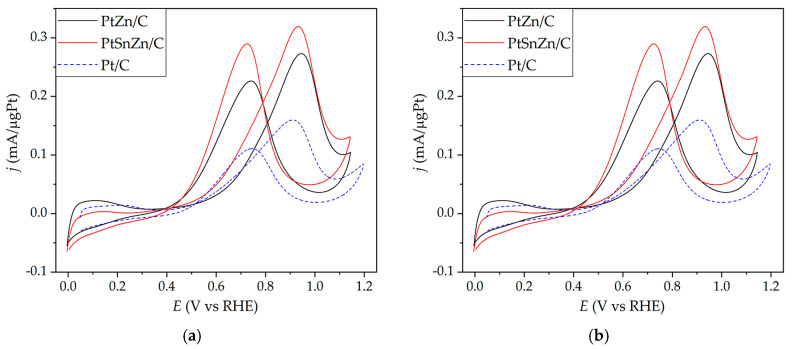
Potentiodynamic curves for the oxidation of 0.5 M CH_3_OH in 0.5 M H_2_SO_4_, v = 50 mV/s in as-prepared PtZn/C (black line), PtSnZn/C (red line) and Pt/C (blue dot line) catalysts. Catalyst activities are expressed according to the real surface area (**a**) and mass of platinum (**b**) of the synthesized catalysts.

Therefore, efficient removal of CO_ads_ will greatly impact the activity of methanol oxidation activity. As can be seen from Figure 5, the beginning of methanol electrooxidation for the synthesized catalysts is in the region where hydroxyl ion adsorption occurs (~0.4 V vs. RHE), so good values for catalyst activity can be expected [43]. The reaction is two times increased at the PtSnZn/C catalyst in comparison to Pt/C and PtZn/C catalysts when the activity is observed according to the real surface area (Figure 5a). When looking at the activity expressed according to the mass of platinum, a significant increase in activity can also be seen for the PtSnZn/C catalyst compared to the Pt/C catalyst and PtZn/C catalyst (Figure 5b). However, from Figure 5b, we can see a more significant increase in the activity of the PtZn/C catalyst compared to the Pt/C catalyst, which supports the fact that the addition of zinc to the platinum catalyst also has an effect on increasing the activity for the methanol oxidation reaction. In comparison to the Pt/C catalyst synthesized by exactly the same procedure, both PtZn/C and PtSnZn/C catalysts show very slightly shifted onset potentials for methanol oxidation. Considering that during the oxidation of adsorbed CO, it was shown that the reaction on PtSnZn/C starts at the earliest potentials, an increase in the activity for the methanol oxidation reaction of this catalyst is quite expected. However, it can be seen from Figure 5 that the activities of methanol oxidation at low potentials on all synthesized catalysts are very similar, although one would expect, based on the direct oxidation of CO on the PtSnZn/C catalyst, an increase in activity in this potential range. This is a consequence of the fact that the adsorption and dehydrogenation of methanol require more Pt atom sites.

In addition to the onset potentials, two other very important indicators that testify to the efficiency of the catalyst are the maximum peak currents in the forward scan (j_max_) and the ratio between forward and reverse peak currents (j_f_/j_b_). The maximum current densities for the methanol oxidation reaction are 0.48 mA/cm^2^ for PtZn/C and 0.99 mA/cm^2^ for PtSnZn/C catalysts. Compared to the PtSn/C catalyst synthesized by the same procedure, the PtSnZn/C catalyst shows better activity [44]. Since the backward oxidation peak represents the removal of unwanted intermediates that are formed during the forward scan, the j_f_/j_b_ ratio can be an important parameter that indicates catalysts’ tolerance to CO_ads_ poisoning [45,46,47]. Compared with commercial Pt/C E-TEK catalysts (j_f_/j_b_ = 0.99), synthesized PtZn/C and PtSnZn/C catalysts also show higher values. It can be concluded that Sn has exceptional importance in the activity of the methanol electrooxidation reaction. Nevertheless, the nanostructuring of Pt with Sn and Zn results in even greater activity, which was the goal of this work.

It should also be noted that according to studies, the ideal size of PtZn particles required for effective catalytic activity is below 4 nm [24,25]. The results of the TEM analysis show that the particle size of PtZn/C and PtSnZn/C catalysts is below 2 nm, which additionally contributes to the high catalytic activity in the methanol electrooxidation reaction. The exceptional contribution of the synthesized catalysts can be observed by comparing the mass activities of the synthesized catalysts with the mass activity of the Pt/C catalyst. By doping platinum with Sn and Zn, we can design advanced Pt-based catalysts for the purpose of improving MOR performance.

**Figure 6:**
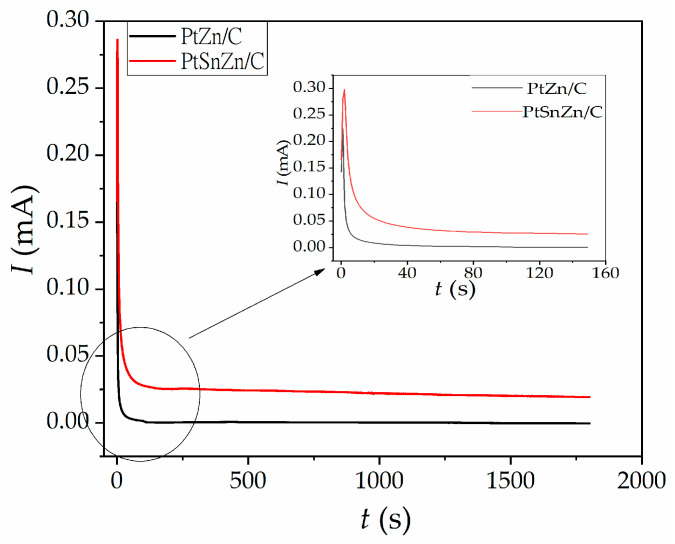
Chronoamperometric curves for the oxidation of 0.5 M CH_3_OH in as-prepared PtZn/C (black line) and PtSnZn/C (red line) catalysts in H_2_SO_4_/CH_3_OH solution at 0.2 V vs. SCE. Inset: enlarged part of the chronoamperometric curves with a time interval range from 0 to 150 s.

The mass activity for PtZn/C is ~1.6, and for PtSnZn/C catalyst, it is ~2 times greater in comparison to Pt/C catalyst with the same platinum loading. This implies that synergistic effects are achieved between Pt, Sn and Zn atoms. The performance of PtZn/C and PtSnZn/C catalysts are also benchmarked with commercial Pt/C E TEK catalysts, which are also proving better electrochemical activity [48]. In order to test the electrocatalytic stability of the catalysts, chronoamperometric measurements were performed in H_2_SO_4_/CH_3_OH solution. Chronoamperometric curves for as-prepared PtZn/C and PtSnZn/C catalysts are displayed in Figure 6.

Both catalysts demonstrate very similar behavior. At the start of the examination, the current drops sharply and then decreases very slightly. For the PtSnZn/C catalyst, the current stabilizes at a higher value than for the PtZn/C catalyst proving better tolerance to catalysts deactivation during the methanol oxidation reaction. In order to more clearly present the currents in the chronoamperometric curves for PtZn/C and PtSnZn/C catalysts (Inset of Figure 6), a shortened range of the time interval is shown. The results of the chronoamperometric stability test support our earlier published results in which the Pt/C catalyst is synthetized by a microwave-assisted method on the carbon support [44].

Based on all the electrochemical tests related to the catalyst efficiency for MOR, the performances of the PtZn/C and PtSnZn/C catalysts were compared with the corresponding catalysts found in the literature and shown in Table 3.

## 4. Conclusions

This work opens a new direction for the synthesis of platinum-based nanoparticles for enhanced MOR in fuel cell applications. Carbon-supported PtZn and PtSnZn nanocatalysts were synthesized successfully by the microwave-assisted polyol procedure. The notable electrocatalytic performances of PtZn/C and PtSnZn/C catalysts are provided by their impressive physical-chemical properties, such as nanoparticle sizes, structure, high surface areas and chemical properties. The number of metal nanoparticles deposited on the carbon substrate was calculated to be ~20 mass % according to the TGA, which proves a successful synthesis procedure without metal loss. TEM examination of all catalysts confirmed uniform dispersion particles over carbon support and nanoparticle diameters of 1.63 ± 0.3 for PtZn/C and 1.8 ± 0.4 for PtSnZn/C catalyst. EDS analysis showed the presence of Zn and Sn in the platinum catalyst, indicating that the obtained ratio of metals differs slightly from the desired values. Further, effective methanol oxidation, high electrocatalytic activity, negative shift of the onset potential, great tolerance to poisoning species and satisfactory stability are also demonstrated. In comparison to the corresponding catalyst with the same metal loading (Table 3), we can conclude that the PtZn/C catalyst has a similar and even ~2 times better mass activity. It should also be noted that the PtZn/C catalyst shows better activity compared to the same synthesized Pt/C catalyst. To the best of our knowledge, the MOR activity of the PtSnZn/C catalyst has not been reported in the literature so far. In comparison to Pt/C catalysts, synthetized PtSnZn/C catalysts have shown 2 times better mass and specific activity. Synthetized PtZn/C and PtSnZn/C catalysts also showed 2 times better activity than commercial Pt/C J.M. Tanaka catalysts [49]. A notable advance in the field of designing PtZn/C and PtSnZn/C catalysts for the methanol oxidation reaction lies in the well-designed trimetallic structure of the catalyst and shows a way of economically using the noble metal. With better MOR activity and lower cost compared to commercial Pt catalysts and catalysts found in the literature, PtSnZn/C and PtZn/C catalysts open the possibility of reducing the amount of expensive noble metals in DMFC.

## Figures and Tables

**Table 1:** The results of TGA and EDS analyses of PtZn/C and PtSnZn/C catalysts.

Catalyst	Metal Content from TGA (Mass %)	The Pt:Zn and Pt:Sn:Zn Atomic Ratios	
		Nominal (Mass %)	EDS (Mass %)
PtZn/C	27.68	75:25	87:13
PtSnZn/C	22.87	63:21:12	70:21:9

**Table 2:** The electrochemically active surface area (ECSA) of PtZn/C, PtSnZn/C and Pt/C catalysts calculated from CO_ads_ stripping voltammetry.

Catalyst	PtZn/C	PtSnZn/C	Pt/C
ECSA (cm^2^)	1.77	1.01	3.02

Since CO does not adsorb at Sn and Zn [40], the surface area for the catalyst refers only to Pt.

**Table 3:** Summary of maximum forward activity, j_f_/j_b_ expressed in mass and specific activity for synthesised PtZn/C, PtSnZn/C and catalyst that can be found in the literature.

Catalyst	Maximum forward Activity (mA/µg Pt)	j_f_/j_b_ from Mass Activity	Maximum forward Activity (mA/cm^2^)	j_f_/j_b_ from Specific Activity	Ref.
PtZn/C	0.27	1.17	0.48	1.11	This work
PtSnZn/C	0.32	1.11	0.99	1.26	This work
Pt/C	0.16	1.33	0.45	1.53	This work
Pt/C E Tek (20% Pt)	0.1	1.10	/	/	[49]
PtSn/C	/	/	0.8	1.09	[44]
PtZn/C	0.22	1.46	/	/	[47]
PtZn/C CNT	0.18	1.2	/	/	[10]

## Data Availability

The data presented in this study are available on request from the corresponding author or co-authors. The data are not publicly available.

## Supplementary Materials

The following supporting information can be downloaded at: https://www.mdpi.com/article/10.3390/ma16134617/s1. Figure S1: TGA curve for PtZn/C catalyst; Figure S2: TGA curve for PtSnZn/C catalyst; Figure S3: EDS analysis of PtZn/C catalyst; Figure S4: EDS analysis of PtZn/C catalyst.
