# Supplementary material for: Design of Pt-Sn-Zn Nanomaterials for Successful Methanol Electrooxidation Reaction
Source: Materials (Basel). 2023 Jun 27;16(13):4617. doi: 10.3390/ma16134617 (PMC10342477; doi:10.3390/ma16134617)
Supplement: Supplementary file 1 [file materials-16-04617-s001.zip › materials-2446866-supplementary.pdf]

Supplementary 1

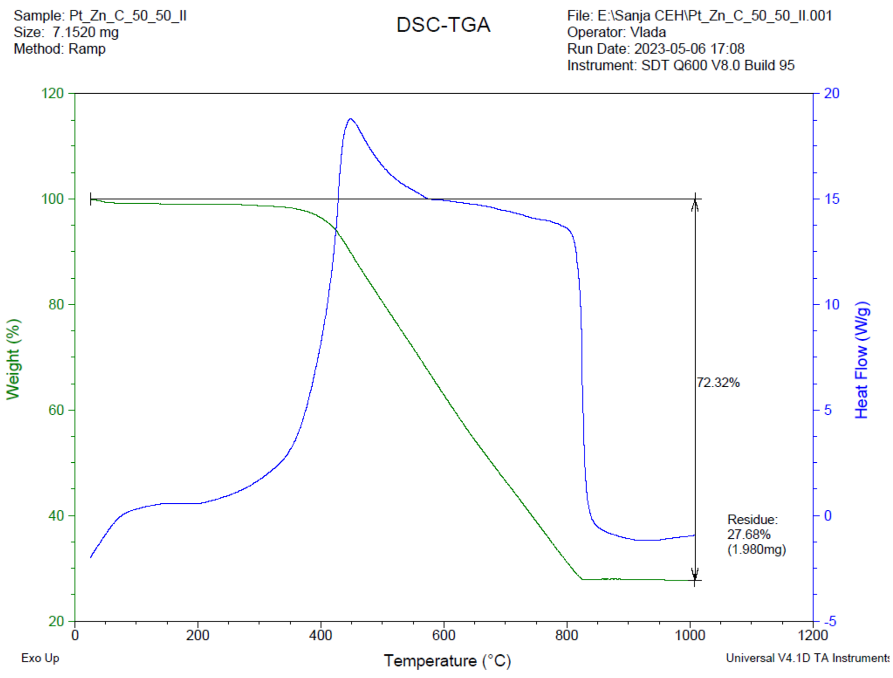

Fig S1. TGA curve for PtZn/C catalyst

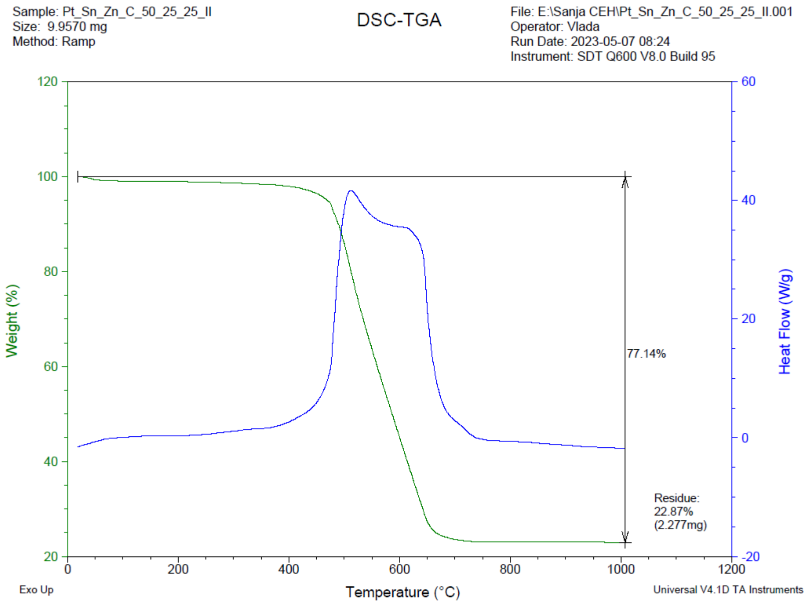

Fig S2. TGA curve for PtSnZn/C catalyst

Supplementary 2

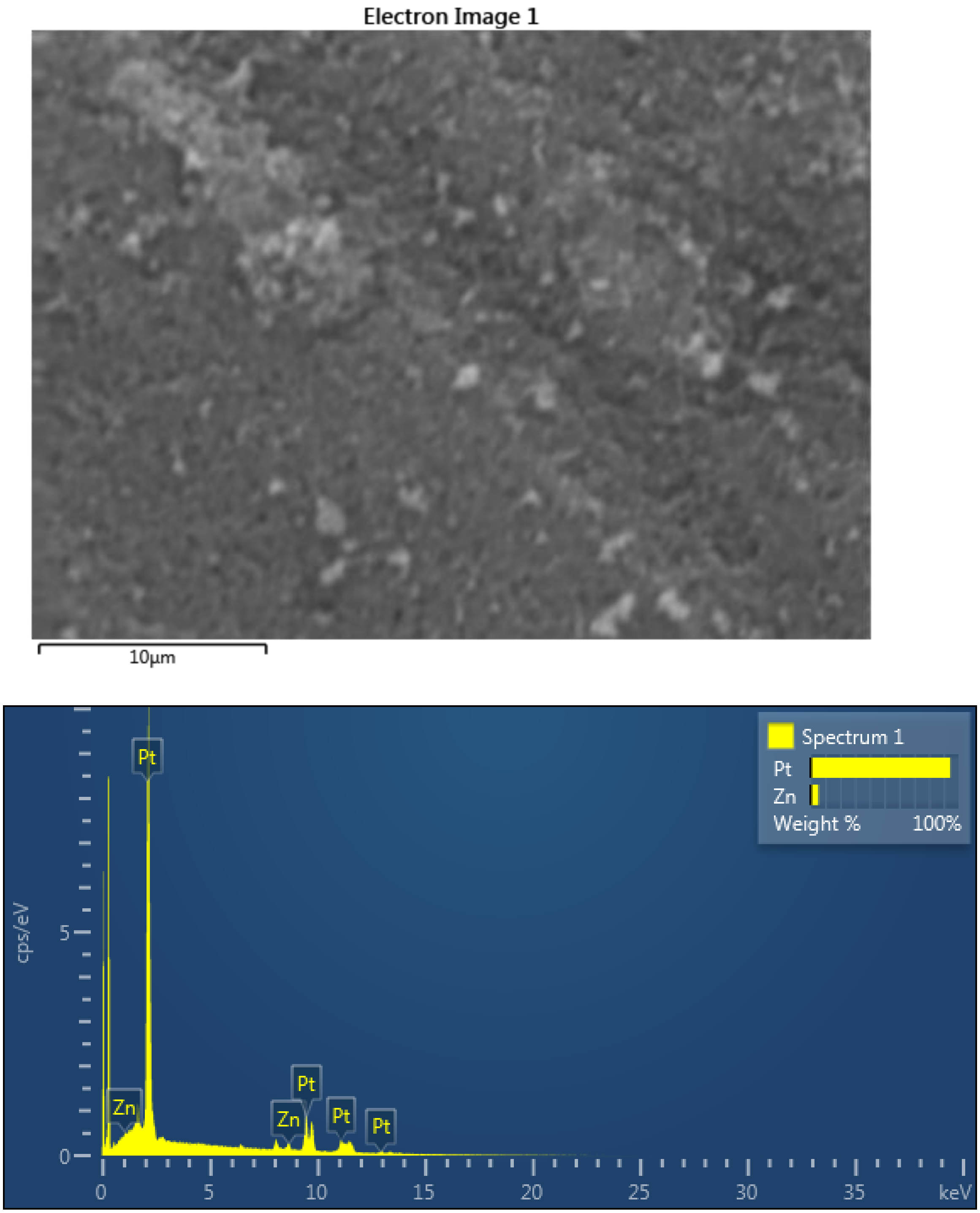

Fig S3. EDS analysis of PtZn/C catalysts

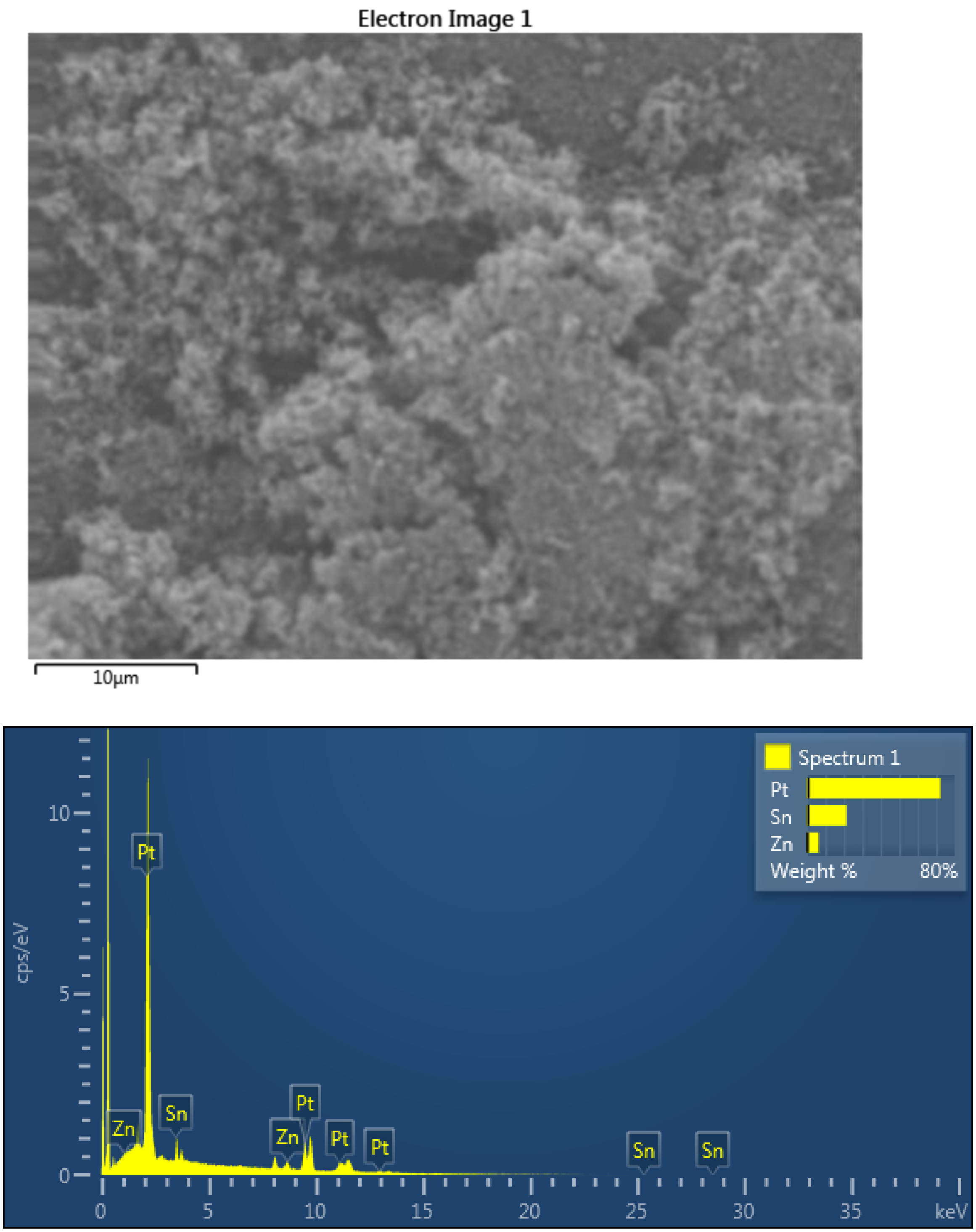

Fig S4. EDS analysis of PtSnZn/C catalysts
